# Study on the Photothermal Performance of a “Thermal Shielding” Coating Using Tungsten Bronze as Functional Material for Asphalt Pavement

**DOI:** 10.3390/ma16227150

**Published:** 2023-11-14

**Authors:** Ling Zhang, Pan Ding, Wei Si, Xingxiang Kang, Hongfei Zhang, Qiutai Gu

**Affiliations:** 1 Lanzhou Highway Development Center, Lanzhou 730000, China; 2022121195@chd.edu.cn (L.Z.); 2018903111@chd.edu.cn (P.D.); 2 Key Laboratory for Special Area Highway Engineering of Ministry of Education, Chang’an University, Xi’an 710064, China; siwei@chd.edu.cn; 3 Highway Development Center of Dongtai City, Yancheng 224200, China; kangxingxiang@chd.edu.cn; 4 China State Construction Silk Road Investment Group Co., Ltd., Xi’an 710075, China; 2020221293@chd.edu.cn

**Keywords:** highway engineering, asphalt pavement, coating, non-stoichiometric compound containing tungsten, photothermal performance

## Abstract

Asphalt pavements absorb more than 90% of the incident solar radiation, which induces not only high-temperature degradation but also the urban heat island (UHI) effect. In this study, a novel nanoscale non-stoichiometric compound containing tungsten (M_x_WO_3_) was used for the first time to prepare thermal shielding coatings to reduce the temperature of pavements and mitigate the UHI effect. Coatings with good shielding characteristics were selected for outdoor thermal insulation tests to evaluate their properties. M_x_WO_3_ (M = K, Na, Cs) exhibited significant thermal shielding, especially Cs_x_WO_3_. Outdoor thermal insulation tests were performed for the Cs_x_WO_3_ coatings, and it was found that the greater the doping, the more significant the thermal shielding effect. Compared with untreated pavements, the surface-coated pavement exhibited significant cooling at 5 cm and 15 cm depth-wise, which reduced the overall pavement temperature by 1–2 °C, and the coating thickness affected the cooling effect.

## 1. Introduction

**Figure 1:**
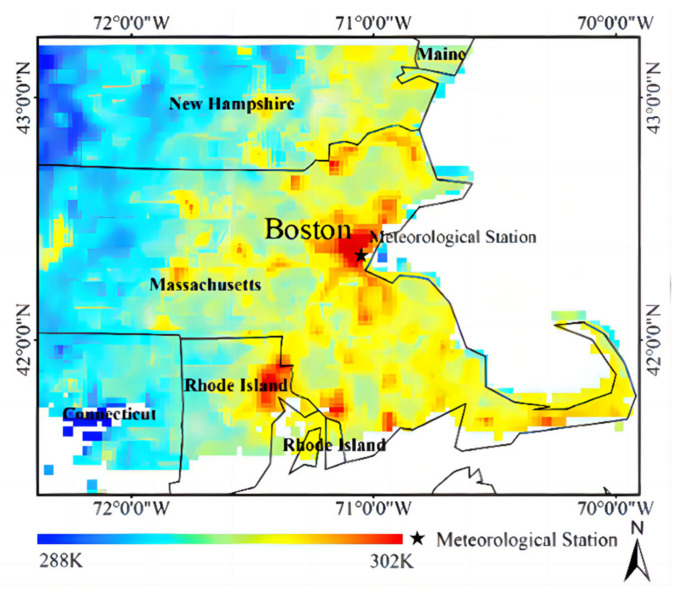
Instance map of the UHI effect [10].

**Figure 2:**
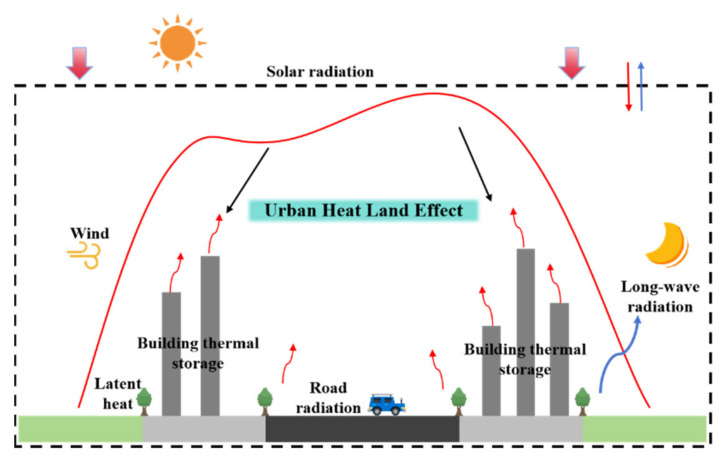
Schematic illustrating the UHI effect.

Black asphalt pavements, which have a high solar radiation-absorbing capacity and strong heat storage capabilities, often experience excessively high temperatures under intense solar radiation. High temperatures lead to an increased occurrence of pavement distress, such as rutting, and contribute to a higher urban heat island effect [1,2,3,4]. Consequently, this issue has garnered significant attention in both the fields of engineering and environmental science, as shown in Figure 1 and Figure 2.

To mitigate the aforementioned high-temperature-related issues, researchers have proposed new types of asphalt pavements, including water-retaining pavement, PCM-impregnated pavement, reflective pavement, high-conductive pavement, and energy-harvesting pavement [5,6,7,8]. Compared to traditional dense asphalt concrete, these new or unconventional asphalt pavements exhibit lower surface temperatures throughout the day or during certain time periods. From an environmental science perspective, whether these new pavement structures effectively alleviate the urban heat island effect remains a subject of study [9]. However, from an engineering technology standpoint, the reduction in extremely high temperatures on pavement surfaces does indeed contribute to a decreased likelihood of high-temperature pavement damage [3,4].

Among the types of pavement mentioned above, reflective pavement is considered to be a type of asphalt pavement with limited application restrictions that can effectively mitigate the urban heat island effect [7]. Surface gritting with light-colored aggregate, chip seals with light-colored aggregate, sealing, resurfacing, coatings and pigments, and other methods can increase the reflectivity of pavement surface [9,11]. In recent years, with the rapid development and wide application of organic resin materials and nanomaterials, pavement reflective coating has been the most preferred technique for reflective pavement, with merits such as excellent and controllable optical properties, simple construction, strong adhesion to asphalt pavement, and no adverse effects on the mechanical properties of asphalt mixtures being proven [12,13,14].

Numerous studies have reported a significant cooling effect of reflective coatings on asphalt pavements. However, as research on reflective pavements continues to advance, limitations in their application have become evident, especially on urban roads surrounded by dense buildings [15,16,17,18]. A simulation study conducted by Yaghoobian et al. in Phoenix, Arizona, showed that when the ground surface reflectivity increases from 0.1 to 0.5, the annual cooling load of a local four-story office building increases by 11% (33.1 kWh·m^−2^) [19]. In addition, the increase in heating energy consumption in winter caused by the use of reflective pavement is likely to be greater than the cooling energy demand saved in summer [20]. Hence, preserving the benefits of pavement coatings and replacing reflective functional materials with new ones to develop novel asphalt pavement cooling coatings that are better suited for urban roads is a promising scientific endeavor.

In recent years, new nanoscale M_x_WO_3_ materials have been widely used in the fields of construction materials and insulating glass [21]. Local surface plasmon resonance [22] and small-polaron absorption [23] have resulted in good near-infrared thermal shielding properties, wherein infrared radiation was absorbed from 700 to 2500 nm, which is an ideal thermal shielding material [24] and has already demonstrated its excellent cooling performance [25,26]. Therefore, in this study, we exploit the use of nano-M_x_WO_3_ for road engineering as a novel functional material for the development of a “thermal shielding” coating. The coatings were tested using a UV/VIS/NIR spectrophotometer (PerkinElmer Lambda 950) to evaluate the effect of different cations (M = Na, K, Cs) and doping levels (2%, 4%, and 6%) of M_x_WO_3_ on the optical properties of the road coatings to achieve excellent near-infrared light-shielding and to test their thermal insulation effect under actual outdoor solar radiation conditions.

## 2. Materials and Methods

### 2.1. Raw Materials

#### 2.1.1. Functional Fillers

The complex crystal structure of M_x_WO_3_ (cubic, tetragonal, and hexagonal phases) results in excellent characteristics, including near-infrared light-shielding properties. In its structural formula, x is in the range of 0 to 1, and M can be an alkali metal, an ammonium ion, or a rare earth metal ion. The properties of M_x_WO_3_ vary for different cations. In this study, three tungsten-containing non-integrable compounds, Cs^+^, Na^+^, and K^+^, were selected. The three types of tungsten bronze nanoparticles used in this study are all of reagent grade and were purchased from Hangzhou Jikang New Materials Co., Ltd., Hangzhou, China.

#### 2.1.2. Binders and Additives

The epoxy resin and chemical additives (curing agent and toughening agent) were used as film-forming substances, and the specific parameters of the reagents are listed in Table 1, Table 2 and Table 3. The coating’s constituent materials are all of reagent grade and were purchased from Shanghai Q.z. New Mates Tech Co., Ltd., Shanghai, China.

### 2.2. Preparation of the Coatings

**Figure 3:**
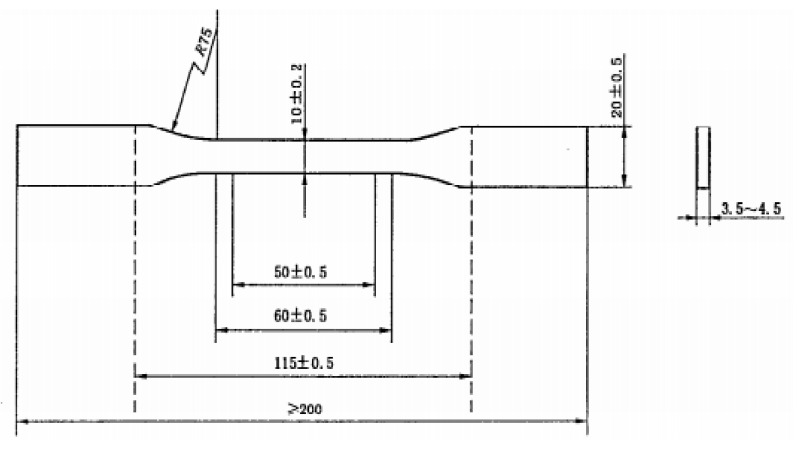
Dimensional drawing of the road coating mould.

**Figure 4:**
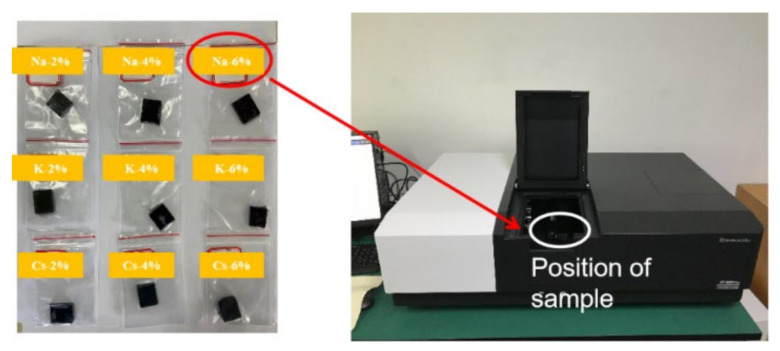
UV/VIS/NIR spectrophotometer test device and photograph of the samples.

First, the epoxy resin with a fixed ratio of the toughening agent was mechanically stirred for 5 min at a rate of 100–150 r/min to obtain mixture A. Second, a curing agent was added to mixture A, and mechanical stirring was conducted for 10 min at a rate of 150–200 r/min to obtain mixture B. Finally, the tungsten bronze nanoparticles were blended into mixture B and mechanically stirred for 10 min at a rate of 500–1000 r/min to obtain mixture C. (Table 4 presents the components of each coating in this study along with their corresponding abbreviations.) Mixture C was slowly poured into a mould that was coated with a release agent, and its size is shown in Figure 3. After casting was completed, the mould was maintained at room temperature (25 °C) for 24 h; the mould was then released, and the coated specimen was obtained. The optical properties of the coating were tested using a 2 cm × 2 cm sample, as shown in Figure 4.

### 2.3. Experiment Methods

#### 2.3.1. Functional Filler Performance Characterization

In this study, three types of tungsten bronzes with good infrared shielding properties, Na_x_WO_3_, K_x_WO_3_, and Cs_x_WO_3_, were used to prepare the coatings. X-ray diffraction (XRD) and UV/VIS/NIR spectrophotometer were employed to evaluate the purity, crystallisation, and optical properties of M_x_WO_3_.

#### 2.3.2. Optical Properties of Road Coatings

The absorbance of the coating was measured according to the ASTM E903-12 standard [27]. The measurement device was a UV/VIS/NIR spectrophotometer (Perkin Elmer Lambda 950 with accuracy ± 0.08 nm) equipped with an integrating sphere (150 mm diameter). The measurement interval was set to 1 nm. The average absorbance value was obtained based on three records for each sample.

#### 2.3.3. Tests on the Thermal Insulation Effect of the Pavement Coatings

**Figure 5:**
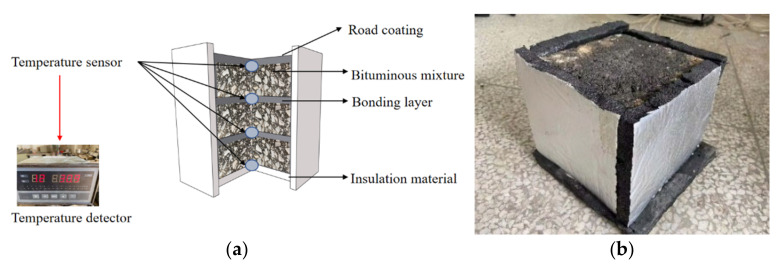
Schematic of the test device for the insulation effect of pavement coatings and the arrangement of the temperature sensors. (**a**) Diagram of the internal structure for testing the temperature of an insulated specimen; (**b**) Physical view of the test insulation specimen.

A 300 mm ×300 mm ×50 mm asphalt slab was prepared and cut into four portions (150 mm × 150 mm × 50 mm). Three of them were stacked, and four temperature sensors were placed at the depth of 0, 5, 10, and 15 cm from the surface of the asphalt slabs (The four positions are denoted as P_0_, P_5_, P_10_, and P_15_, respectively), and the specimens were wrapped with insulation material around the perimeter and bottom. The surface of the test pieces was coated with different pavement coatings and placed outdoors without any shelter, and the insulation effect of the coating was analyzed based on the temperature profile obtained using a temperature patrol, as shown in Figure 5. In this study, we used PT100 thermocouple temperature sensors with a resolution of 0.1 °C, and data were collected at 5 min intervals.

### 2.4. Data Analysis Methods

To better evaluate the test results of the optical properties of the coatings with different schemes, data standardization (normalization) was used to process the raw data to address the issue of comparability between the data.

In this study, min–max normalization was used to linearly transform the raw data so that the resultant values were mapped between [0–1]. The transformation function is given by Equation (1):

(1) $$x^{*}=\frac{x-\min}{\max-\min}$$

where max and min are the maximum and minimum values of the data, respectively.

## 3. Results

### 3.1. Performance Analysis of M_x_WO_3_

#### 3.1.1. Results of XRD

**Figure 6:**
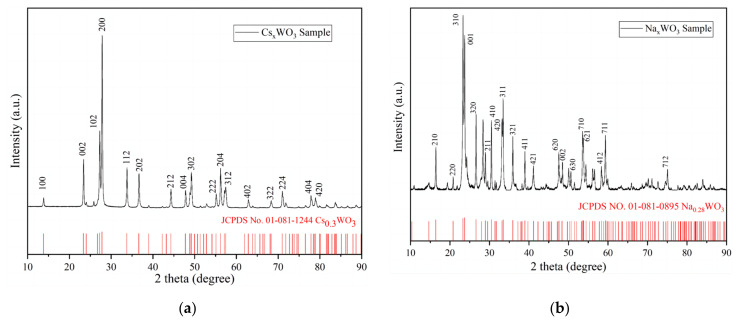

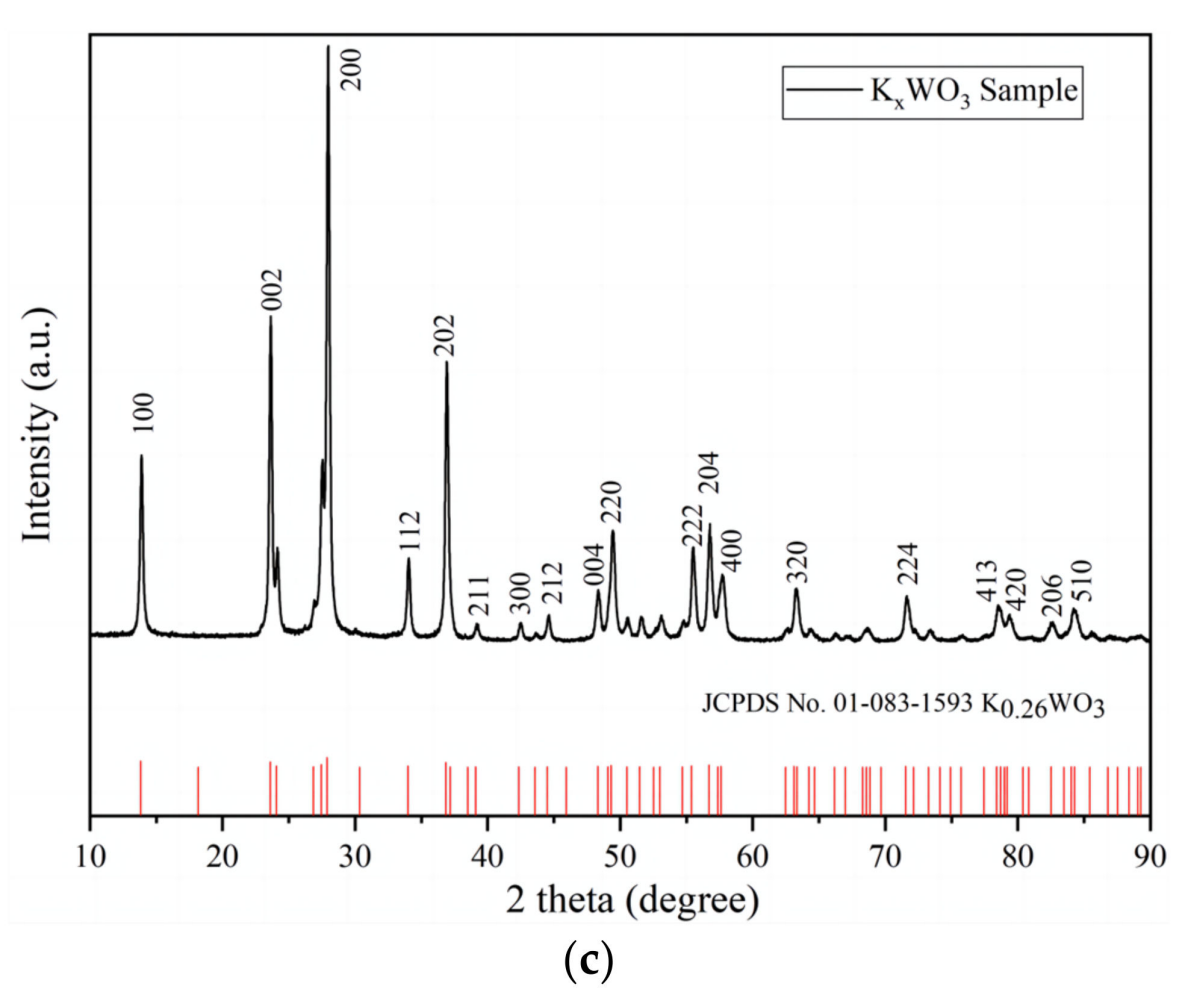
XRD test results (red lines are the standard reference) of M_x_WO_3_ functional fillers. (**a**) Cs_x_WO_3_; (**b**) Na_x_WO_3_; (**c**) K_x_WO_3_.

XRD measurements were employed to evaluate the purity and crystallisation of the M_x_WO_3_ (M = K, Na, Cs) material used in this study. The XRD spectrum of M_x_WO_3_ is displayed in Figure 6, along with the corresponding standard XRD patterns and their JCPDS numbers at the bottom of each figure. The samples that best match the overall diffraction peaks with K_x_WO_3_, Na_x_WO_3_, and Cs_x_WO_3_ are the hexagonal K_0.26_WO_3_ (JCPDS NO. 01-083-1593), tetragonal Na_0.26_WO_3_ (JCPDS NO. 01-081-0895), and phase Cs_0.3_WO_3_ (JCPDS NO. 01-081-1244) standard reference materials, respectively.

Comparing the diffraction peaks of the standard reference materials with those of the samples, it is evident that the diffraction peaks with higher relative intensity in the standard materials correspond well to the sample’s diffraction patterns. Additionally, some weak diffraction peaks are present in the sample that do not match the peaks of the standard reference materials, indicating the presence of some crystalline phases (impurities) in the sample that differ slightly from the standard materials. However, the relative intensities of these peaks are extremely small, demonstrating their minimal presence in the sample. Based on this, it is reasonable to conclude that the three samples used in this study are of high purity and well-formed crystalline tungsten bronze nanoparticles.

#### 3.1.2. Optical Properties of M_x_WO_3_

**Figure 7:**
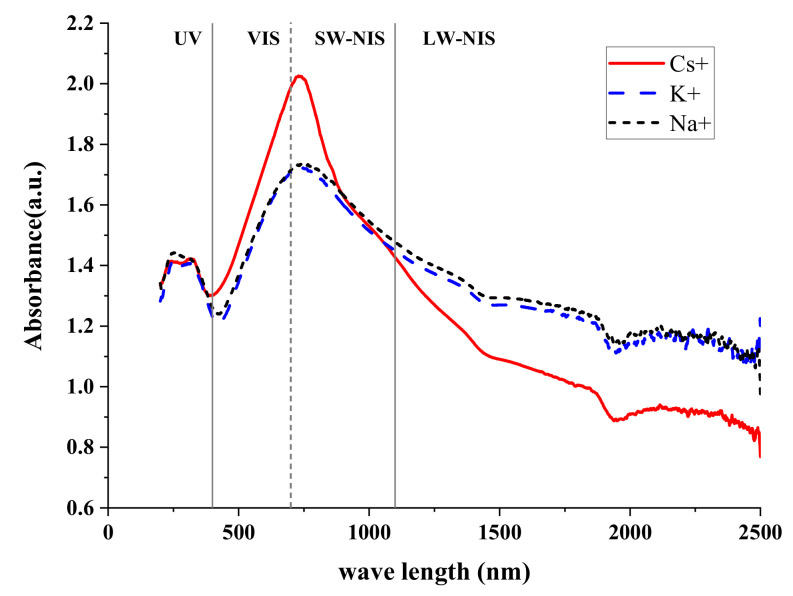
Optical performance of M_x_WO_3_.

Figure 7 and Table 5 shows the absorbance of the three M_x_WO_3_ materials selected for this study. The figure shows that the three functional fillers have significant peaks in the visible and near-infrared region from 400 to 1100 nm. It is known that 70–90% of solar energy is concentrated in this region [28]. This indicates that all three selected M_x_WO_3_ (M = K, Na, Cs) materials have good NIR shielding properties. The NIR shielding performance of Cs_x_WO_3_ was found to be the best.

### 3.2. Optical Properties of Road Coatings

**Figure 8:**
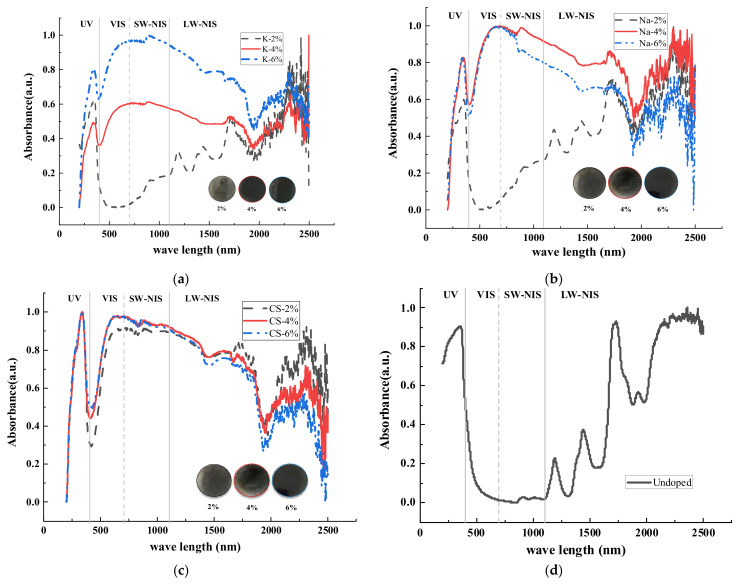
Absorbance of coatings with and without the addition of tungsten-containing non-stoichiometric compound. (**a**) k_x_WO_3_ doped coatings; (**b**) Na_x_WO_3_ doped coatings; (**c**) Cs_x_WO_3_ doped coatings; (**d**) undoped coating.

The absorbance spectra of the road coatings containing M_x_WO_3_ in the wavelength range of 200–2500 nm are shown in Figure 8 (normalized results). Figure 8a–c show the absorbance spectra of M_x_WO_3_ at doping levels of 2, 4, and 6% and Figure 8d shows the absorbance spectra of undoped coating, respectively. Figure 8 indicates that the type and concentration of M_x_WO_3_ affect the absorbance spectra of the coatings directly; a higher doping level contributes to improved NIR shielding. The NIR shielding of the road coatings using Cs_x_WO_3_ was excellent and similar at concentrations of 2, 4, and 6%.

**Figure 9:**
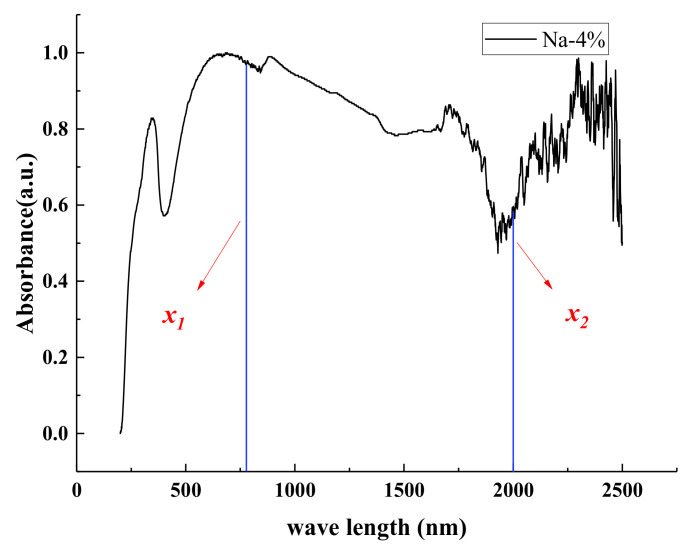
Integration algorithm (Na-4% as an example).

**Figure 10:**
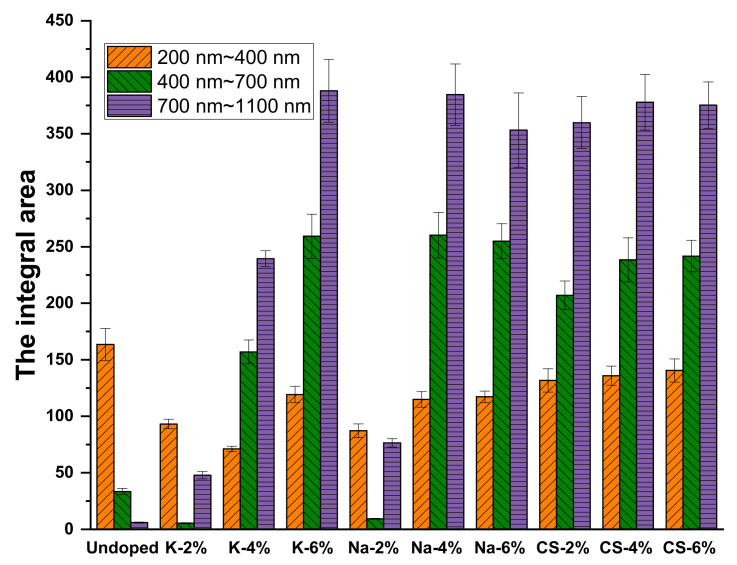
Infrared thermal shielding effect of the coatings.

To further quantify the effects of different M_x_WO_3_ materials and the concentration on the near-infrared thermal shielding properties of the coatings, the absorbance spectral curves were integrated using Na-4% as an example (Figure 9), and the absorbance values of the coating in the infrared region ranging from $x_{1}$ nm to $x_{2}$ nm were integrated according to Equation (2). Figure 10 shows a summary of the integrated area of the coating in the 200–400 nm, 400–700 nm, and 700–1100 nm regions.

(2) $$S=\int_{x_{1}}^{x_{2}}L_{M_{x}WO_{3}}dx$$

where *S* is the area of integration corresponding to the coating in the wavelength range of *x*_1_ to *x*_2_.

*x*_1_ and *x*_2_ represent the upper and lower boundaries of the integration wavelength range, respectively, and $L_{M_{x}WO}{}_{{}_{3}}$ is the absorbance value of the road coatings.

As shown in Figure 10, in the UV region with wavelengths from 200 to 400 nm, the integrated area of the absorbance value profile exhibits varying degrees of attenuation, but the degree is so small that it has a negligible effect. In the visible region spanning wavelengths from 400 to 700 nm, the integrated area of the absorbance value of the road coatings increases significantly with the increased concentration of functional materials, except for the K-2% and Na-2% coatings, which decreased. This occurs because the M_x_WO_3_ (M = Cs) material has a better light absorption capacity at 400–700 nm than M_x_WO_3_ (M = K, Na); when doped with smaller amounts of K_x_WO_3_ and Na_x_WO_3_, the coating solids are transparent, whereas Cs_x_WO_3_ is a dark blue solid powder, and the coating starts to absorb visible light from 2% onwards.

In the short wave near-infrared region spanning wavelengths from 700–1100 nm, the integrated area of light absorbance values corresponding to road coatings doped with functional materials increased substantially, with the K_x_WO_3_ coatings showing a linear increase as the content of functional materials increased. Na_x_WO_3_ exhibit an initial increase followed by a decrease. The spectral absorbance values of coatings containing Cs_x_WO_3_ exhibit very small fluctuations with increasing functional materials dosage and have a good NIR shielding effect. The results indicate that Cs_x_WO_3_ is the best functional material for the preparation of thermal insulation coatings, considering the optical properties of the functional material.

## 4. Discussion

### 4.1. Analysis of the Thermal Insulation Properties of Road Coatings

According to the UV-visible-NIR spectrophotometric analysis, the road coatings prepared from the Cs_x_WO_3_ functional material exhibit excellent optical properties and good light-shielding in the NIR region. Therefore, Cs_x_WO_3_ was selected as the functional material for the preparation of road coatings. Based on a previously published report [29], a dosage of 0.4 kg/m^2^ was applied uniformly to the surface of the specimen between 20 °C and 25 °C. After curing, the specimen was placed in an outdoor environment without any shelter, and the temperature in different layers was monitored using sensors.

#### 4.1.1. Analysis of the Cooling Effect of Coated Pavements

**Figure 11:**
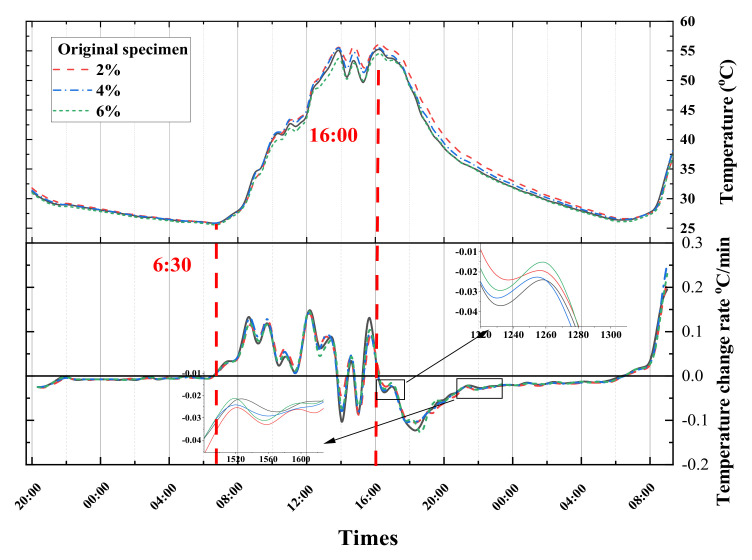

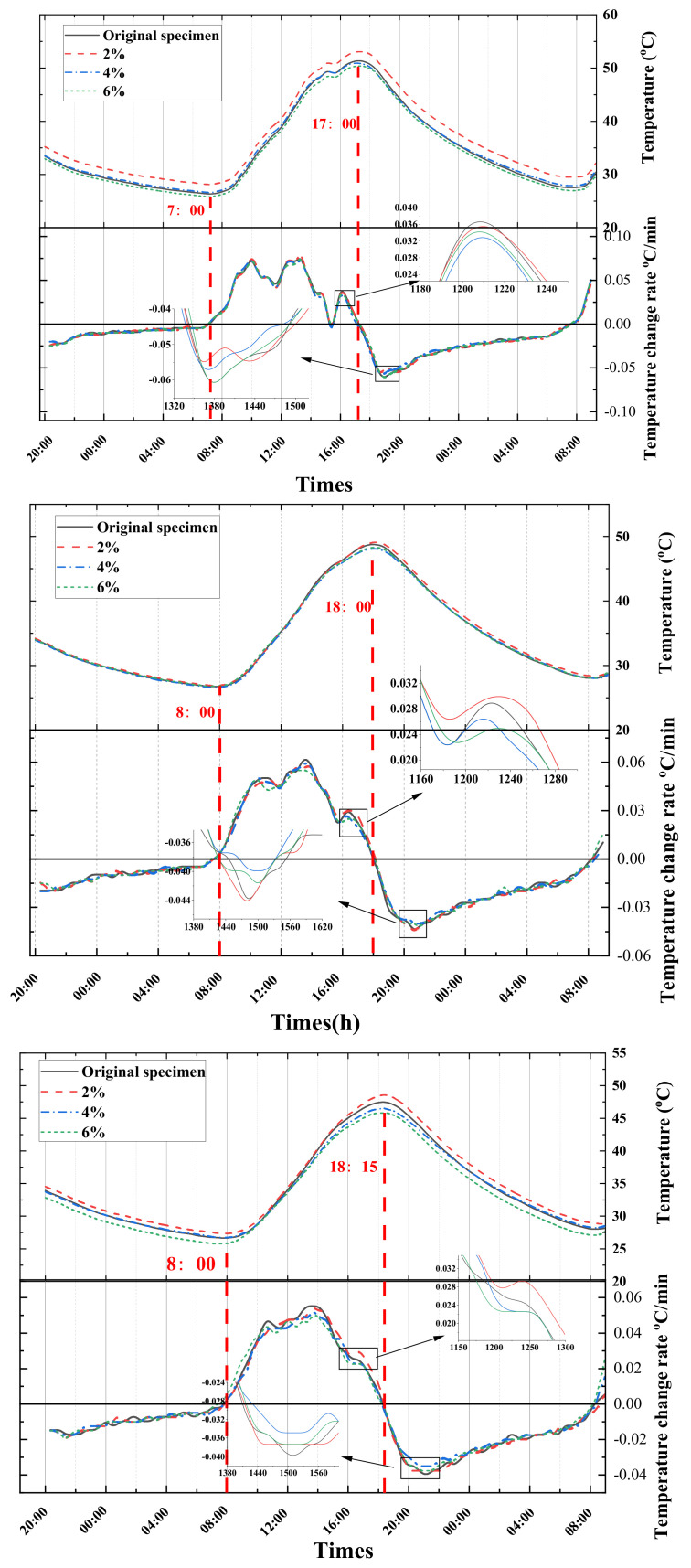
Temperature profile of surface, at the depth of 5 cm, 10 cm and 15 cm from the surface of the specimen (from top to bottom).

Figure 11 show the temperature changes and the corresponding temperature change rate curves for the asphalt slab pieces with road coating of different doping levels (2, 4, and 6% Cs_x_WO_3_ fillers) and without coatings at 0, 5, 10, and 15 cm from their surface in the outdoor environment.

The temperature of different layers has a similar change pattern; for example, with the increasing intensity of solar radiation, the temperature of the pavement structure gradually increases, with the maximum temperatures at the depth of 0, 5, 10, and 15 cm from the surface reaching 56.0, 53.0, 49.0, and 48.5 °C, respectively, which would result in an increased chance of high-temperature degradation such as rutting.

According to the temperature change curves of the different layers, it was found that T_2%_ > T_origin_ > T_4%_ > T_6%_, which shows that the cooling effect of the road coating with 2% Cs_0.3_WO_3_ is not significant, whereas the road coatings of Cs-4% and Cs-6% exhibit thermal insulation effect and reduce the overall temperature of the pavement structure.

According to the temperature change rate curves at the depths of 5, 10, and 15 cm from the surface, HR (heating rate)_origin_, HR_2%_ > HR_4%_ > HR_6%_ in the heating stage, and CR (cooling rate)_origin_, CR_2%_ < CR_4%_ < CR_6%_ in the cooling stage, the road coating can influence the change rate of the pavement temperature by decreasing the heating rate and increasing the cooling rate, thus achieving the effect of “thermal shielding”.

**Figure 12:**
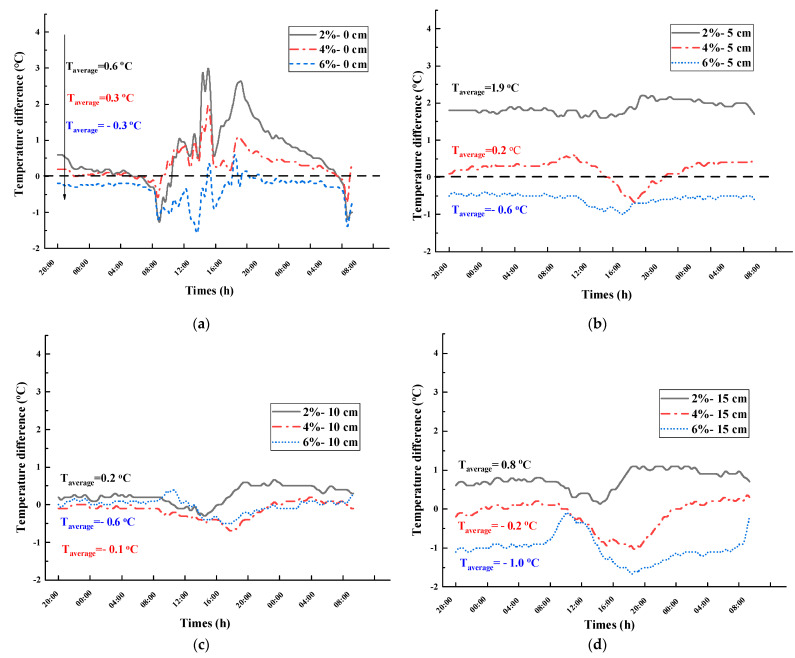
Temperature difference–time variation curves at P_0_, P_5_, P_10_, and P_15_ for different road coatings (27 June 2021). (**a**) Temperature difference curve at P_0_; (**b**) Temperature difference curve at P_5_; (**c**) 10 cm temperature difference curve at P_10_; (**d**) Temperature difference curve at P_15_.

To quantify the thermal insulation effect of the road coating, the temperature of the coated pavement was adjusted to be different from that of the uncoated sample, and the temperature difference curves at P_0_, P_5_, P_10_, and P_15_ were obtained as a function of time, as shown in Figure 12.

From Figure 12, the following are observed:

The doping level is an important factor that affects the thermal insulation effect of the road coating. Taking the temperature difference at P_15_ as an example, the average values of the temperature difference corresponding to the coatings containing 2, 4, and 6% Cs_0.3_WO_3_ are 0.8, −0.2, and −1.0 °C, respectively, which indicates that the thermal insulation effect improves with the doping level.

Comparing the thermal insulation effect in the night cooling stage from 00:00 to 8:00 and the daytime heating stage from 8:00 to 20:00, it is observed that the curve for the former is relatively flat, whereas the curve of the latter shows a downward peak. Moreover, a downward peak of the temperature difference appears at approximately 16:00 to 20:00, which gradually retreats with depth, indicating that the thermal insulation effect of the road coating occurs mainly in the warming phase when the radiation intensity is high.

Comparing the mean values of temperature difference at different depths, the maximum temperature difference at P_0_, P_5_, P_10_, and P_15_ with 6% doping were −1.8, −1.0, −0.5, and −1.7 °C, respectively, with mean values of −0.3, −0.6, −0.6, and −1.0 °C, respectively, indicating that the road coating is more effective at the depth of 5 cm and 15 cm from the surface in the pavement structure.

#### 4.1.2. Analysis of the Thermal Insulation Effect of the Undoped Coating (Matrix without Cs_0.3_WO_3_) Compared to the Road Coating

**Figure 13:**
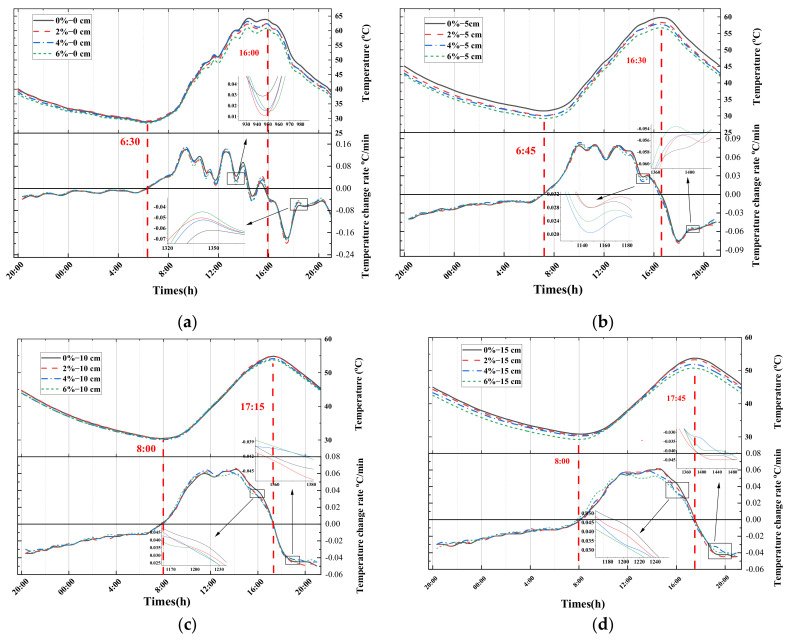
Temperature and temperature rate–time variation curves for different depths at 0, 2, 4, and 6% doping levels (30 June 2021). (**a**) Surface temperature profile of the specimen; (**b**) Temperature profile at P_5_; (**c**) Temperature profile at P_10_; (**d**) Temperature profile at P_15_.

**Figure 14:**
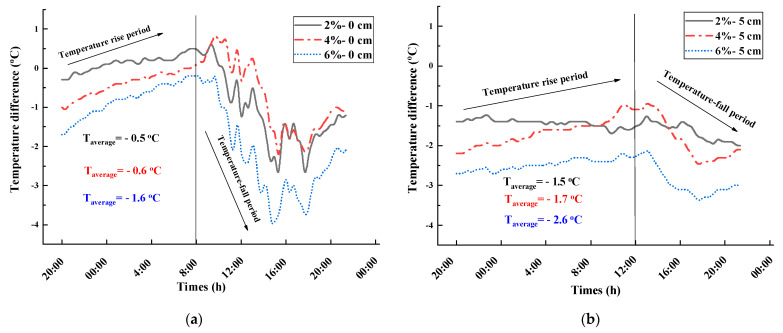

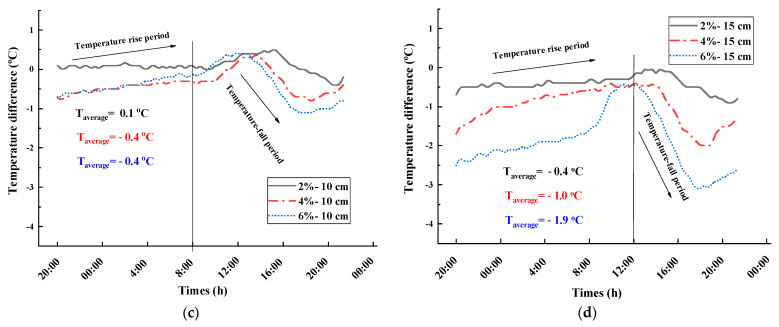
Temperature difference between Cs-2%, Cs-4%, and Cs-6% coated specimens with the undoped coating coated specimen at the same position (30 June 2021). (**a**) P_0_; (**b**) P_5_; (**c**) P_10_; (**d**) P_15_.

The preceding analysis establishes that the road coating reduces the temperature of the pavement structure, but the insulation effect of Cs-2% is poor, especially in P_0_ and P_5_, where the phenomenon of heat storage occurs and the temperature increases. To analyze the cause of this behaviour, a dosage of 0.4 kg/m^2^ without Cs_x_WO_3_ was applied to the surface of the blank asphalt pavement as a control group, corresponding to the analysis in Section 4.1.1. The temperature and temperature difference curves at 0, 2, 4, and 6% doping levels of the road coating at P_0_, P_5_, P_10_, and P_15_ are shown in Figure 13 and Figure 14, respectively.

Figure 13 shows that the trends in both the heating and cooling rates of the pavement structure at different depths are consistent with Figure 14, but the overall pavement structure has different degrees of temperature reduction at different pavement depths for road coatings with doping levels of 2, 4, and 6% compared to the undoped coating; the higher the doping level, the more significant the reduction in the pavement temperature.

Secondly, a quantitative assessment of the thermal insulation effect of the road coatings based on the temperature difference versus time presented in Figure 14 reveals the following:

- At P_5_, the average temperature reduction values of the road surface with coatings containing doping levels of 2, 4, and 6% are 1.5, 1.7, and 2.6 °C, respectively, compared with the undoped coating coated specimen. The greater the amount of functional materials, the more significant the thermal insulation effect.
- Considering Cs-2% as an example, the average values of the temperature difference at P_0_, P_5_, P_10_, and P_15_ are −0.5, −1.5, 0.1, and −0.4 °C, respectively, and the maximum values of the temperature difference were −3.1, −2.0, −0.4, and −0.9 °C, respectively. The most significant thermal insulation effect occurs at P_5_.
- The slope of the temperature difference curve shows a flat slope trend in the cooling stage compared with the temperature difference in the heating stage, whereas the slope is steep in the heating stage. Considering the heat insulation effect of Cs-2% at P_5_ as an example, the average values of the temperature difference in the cooling and heating stages are −1.4 °C and −1.6 °C, respectively. Thus, it is evident that the “thermal shielding” effect of the road coating is more significant in the warming phase.

#### 4.1.3. Analysis of the Effect of Epoxy Resin on the Thermal Insulation Effect of the Coating

Comparing the results of the two thermal insulation tests, it was determined that the epoxy resin, as the matrix of the thermal insulation coating, would affect the thermal insulation effect. The mean and maximum temperature difference of the Cs-2% coated specimen and undoped coating coated specimen are shown in Table 6. The epoxy resin carrier at 0.4 kg/cm^2^ affects the thermal insulation effect of the road coating with a low doping level. Moreover, the inner temperature of the position near the surface appears to increase. This is because the thermal conductivity of the epoxy resin is less than that of the asphalt mixture. In addition, the epoxy resin seals the surface voids of the asphalt mixture after curing. It was determined that coating Cs-2% affects the thermal insulation effect of the pavement structure, compared to undoped coating. This resulted in a reduction of the temperature of pavement at P_0_, P_5_, P_10_, and P_15_ by 3.1, 2.0, 0.4, and 0.9 °C, respectively. The effect of heat insulation and temperature reduction is significant. It was observed that the coating containing M_x_WO_3_ can significantly reduce the temperature of the pavement.

### 4.2. Analysis of the Mechanism of “Thermal Shielding” Road Coatings

**Figure 15:**
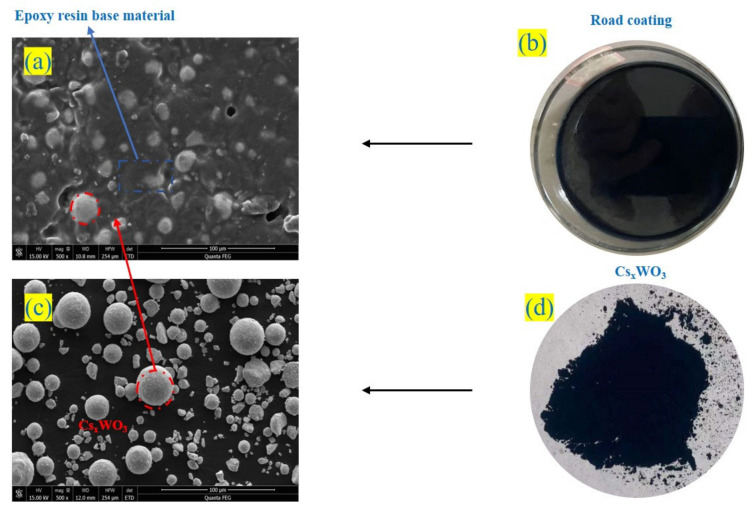
Microscopic morphology of Cs-4%. (**a**) SEM micromorphological image of thermal shielding coating; (**b**) sample of thermal shielding coating; (**c**) SEM micromorphological image of Cs_0.3_WO_3_ functional material; (**d**) sample of Cs_0.3_WO_3_ functional material.

It was determined that the near-infrared light-shielding properties of M_x_WO_3_ materials are due to localized surface plasmon resonance and small-polaron absorption [30,31]. The study revealed that M_x_WO_3_ (M = K, Na, Cs) had good NIR attenuation properties. as determined by spectrophotometric tests. The road coating was produced by homogeneously dispersing M_x_WO_3_ (M = K, Na, Cs) as a functional material in an epoxy resin matrix (Figure 15). Their thermal insulation effect can be attributed to the unique crystal structure inside M_x_WO_3_, which utilizes the heat energy component of solar radiation to reduce hexavalent tungsten to pentavalent tungsten under the action of local plasma resonance and small polaritons, resulting in a thermal shielding effect.

## 5. Economic Analyses

In order to provide readers with insight into this pavement coating from an economic perspective, this section briefly analyzes the cost of coating materials required per square meter of asphalt pavement. The commercial tungsten bronze nanoparticles, epoxy resin E51, curing agent, and toughening agent used in this study are priced at CNY 2000/kg, CNY 30/kg, CNY 50/kg, and CNY 100/kg, respectively. These material prices are based on the 2021 mainland China material market, which is the year of this study. Considering a coating with usage of 0.4 kg·m^−2^ and a tungsten bronze nanoparticle content of 6% for each square meter, the coating requires 0.376 kg of the matrix (including epoxy resin, curing agent, and toughening agent) and 0.024 kg of tungsten bronze nanoparticles. Accordingly, the costs of the matrix and tungsten bronze nanoparticles are CNY 16.2 and CNY 48, respectively. Therefore, the total cost of the coating per square meter is CNY 64.2. It is worth noting that when covering large areas, which involves purchasing materials in bulk, the price of every material used in pavement coating will have certain discounts, further reducing the cost of the coating.

## 6. Conclusions

In this study, the effects of different cations and doping levels on the optical properties and thermal insulation of road coatings were investigated to evaluate the feasibility of using M_x_WO_3_ materials for the preparation of “thermal shielding” road coatings. The main conclusions are as follows:

1. XRD tests showed that the spectra of the three investigated samples of M_x_WO_3_ (M = K, Na, Cs) matched the standard XRD spectra of hexagonal K_0.26_WO_3_, tetragonal Na_0.26_WO_3_, and hexagonal Cs_0.3_WO_3_, respectively. Moreover, the three samples exhibited excellent light-shielding properties in the visible region from 400 nm to 700 nm and in the NIR region from 700 to 1100 nm, especially M_x_WO_3_ (M = Cs).
2. By studying the performance of the road coatings prepared from the three samples of M_x_WO_3_ (M = K, Na, Cs) at 2, 4, and 6% doping levels, it was determined that the thermal insulation effect of the M_x_WO_3_ (M = K, Na) pavement coatings varied significantly with the doping level, and the near-infrared light-shielding characteristics were poor at the 2% doping level, compared to the 4–6% doping level; whereas the coatings with M_x_WO_3_ (M = Cs) had significant NIR shielding properties from 2 to 6%.
3. The most effective Cs_0.3_WO_3_ road coating was used to evaluate the thermal insulation effect in an actual outdoor solar radiation environment, and it was found that the doping level had a significant effect on the insulation characteristics; the greater the doping level, the better the thermal insulation effect. The surface coating of the pavement structure depth-wise at 5 cm and 15 cm from the road surface had a significant cooling effect, and the coating had a good thermal insulation effect on the entire pavement structure, thereby reducing heat accumulation inside the pavement structure. Moreover, by comparing the temperature difference between the daytime warming phase and the night-time cooling phase, it was determined that the “thermal shielding” road coating was effective in the warming phase with high solar radiation levels during the daytime, and the overall temperature reduction of the road surface was 1–2 °C.
4. The epoxy resin used as an insulating coating carrier affects the thermal insulation properties. Although road coatings containing M_x_WO_3_ can significantly reduce the temperature of the pavement structure, the amount or thickness of the coating used influences its cooling effect. In addition, the thickness of the epoxy resin and its thermal properties must be considered when designing “thermal shielding” road coatings.

Through experimental methods, this study has demonstrated that the optical shielding coatings using tungsten bronze nanoparticles as functional materials can effectively reduce extreme high-temperature values in asphalt pavements, albeit with limited impact. Consequently, it is recommended for future research to employ potential techniques, such as surface modification of functional materials and the development of multifunctional composite coatings, to enhance the cooling performance of these coatings. Furthermore, investigating the coating’s durability on the surface of asphalt pavements, including its resistance to wear, aging, and contamination, would be helpful in establishing a clear understanding of how the cooling effect of the coating degrades over time, ensuring long-term stability in its cooling efficiency throughout its service life.

## Figures and Tables

**Table 1:** The basic technical specifications of the curing agent.

Model	Appearance	Active Hydrogen Equivalent	Viscosity at 25 °C (mPa·s)	Density at 25 °C (kg/m^3^)
TMPMP	Colorless, transparent, and low viscous liquid	132.8	100–200	1200–1300

**Table 2:** The basic technical specifications of the toughening agent.

Model	Appearance	Refractive Index at 25 °C (mPa·s)	Viscosity at 25 °C (mPa·s)	Density at 25 °C (kg/m^3^)
CMP-410	Light yellow transparent liquid	1.4450~1.4465	70–80	1000–1020

**Table 3:** Technical specifications of the epoxy resin.

Model	Appearance	Epoxy Value (eq/100 g)	Viscosity at 25 °C (mPa·s)	Density (kg/m^3^)
E-51	Colorless, transparent, and viscous liquid	0.48–0.54	10,000–15,000	1160–1180

**Table 4:** The components of each coating and the corresponding abbreviation.

Number of Coating Sample	The Type of Nanoparticles	Composition of Coatings (wt.% of Coating)		Abbreviation 1.16–1.18
		Nanoparticles	Mixture B	
1	K_x_WO_3_	2	98	K-2%
2		4	96	K-4%
3		6	94	K-6%
4	Na_x_WO_3_	2	98	Na-2%
5		4	96	Na-4%
6		6	94	Na-6%
7	Cs_x_WO_3_	2	98	Cs-2%
8		4	96	Cs-4%
9		6	94	Cs-6%

**Table 5:** Absorbance values at the cut-off point of functional materials.

Functional Materials	Ab_Max_ *	Ab_400nm_	Ab_700nm_	Ab_1100nm_	Integration Area in the 400–1100 nm Region
K	1.722	1.231	1.706	1.448	1076.16
Na	1.735	1.260	1.714	1.477	1091.85
Cs	2.026	1.302	1.988	1.427	1161.22

* Ab_Max_ is the maximum absorbance value; Ab_400nm_, Ab_700nm_, and Ab_1100nm_ are the absorbance values at 400 nm, 700 nm, and 1100 nm, respectively.

**Table 6:** Comparison of the cooling effect of Cs-2% and undoped coating.

Position	Average Value/°C		Optimum Insulation Effect Temperature/°C	
	Test I *	Test II *	Test I	Test II
P_0_	0.6	−0.5	−1.4	−3.1
P_5_	1.9	−1.5	1.6	−2.0
P_10_	0.2	0.1	−0.3	−0.4
P_15_	0.8	−0.4	0.1	−0.9

* Test I represents the temperature difference test between Cs-2% coated specimen and blank asphalt pavement; Test II represents the temperature difference test between Cs-2% coated specimen and undoped coating coated specimen.

## Data Availability

Data will be available upon request.

## Abbreviations

M_x_WO_3_	Tungsten bronze
Cs_x_WO_3_	Cesium tungsten bronze
Na_x_WO_3_	Sodium tungsten bronze
K_x_WO_3_	Potassium tungsten bronze
K-2%	The coating with a K_x_WO_3_ content of 2%; K-4% and K-6% share similar significance
Na-2%	The coating with a Na_x_WO_3_ content of 2%; Na-4% and Na-6% share similar significance
Cs-2%	The coating with a Cs_x_WO_3_ content of 2%; Cs-4% and Cs-6% share similar significance
Undoped coating	The resin matrix without tungsten bronze
P_0_	The surface of the asphalt slabs
P5	The depth of 5 cm from the surface of the asphalt slabs; P_10_ and P_15_ share similar significance
Ab_Max_	The maximum absorbance value
Ab_400nm_	The absorbance values at 400 nm; Ab_700nm_ and Ab_1100nm_ share similar significance
UV	Ultraviolet region spanning wavelengths from 200 nm to 400 nm
VIS	Visible region spanning wavelengths from 400 nm to 700 nm
SW-NIR	The short-wavelength near-infrared region spanning wavelengths from 700 nm to 1100 nm
LW-NIR	The long-wavelength near-infrared region spanning wavelengths from 700 nm to 1100 nm
T_2%_	The temperature at a specific position of the specimen coated with Cs-2%; T_4%_ and T_6%_ share similar significance
T_origin_	The temperature at a specific position of the control specimen without coating
HR	Heating rate
CR	Cooling rate
